# Morning Salivary Cortisol Has a Positive Correlation with GAD-7 Scores in Patients with Ulcerative Colitis

**DOI:** 10.3390/jcm13226707

**Published:** 2024-11-08

**Authors:** Cristina M. Castro, Aditya Mithal, Tina Deyhim, Loren G. Rabinowitz, Olawande Olagoke, Steven D. Freedman, Adam S. Cheifetz, Sarah K. Ballou, Konstantinos Papamichael

**Affiliations:** 1 Department of Medicine, Beth Israel Deaconess Medical Center, Harvard Medical School, Boston, MA 02215, USA; cristina.castro.rivera@gmail.com (C.M.C.); amithal@bidmc.harvard.edu (A.M.); 2 Division of Gastroenterology and Hepatology, Beth Israel Deaconess Medical Center, Harvard Medical School, Boston, MA 02215, USA; tdeyhim@bidmc.harvard.edu (T.D.); lrabinow@bidmc.harvard.edu (L.G.R.); oolagoke@bidmc.harvard.edu (O.O.); sfreedma@bidmc.harvard.edu (S.D.F.); acheifet@bidmc.harvard.edu (A.S.C.); sballou@bidmc.harvard.edu (S.K.B.)

**Keywords:** inflammatory bowel disease, Crohn’s disease, ulcerative colitis, cortisol, anxiety, depression, quality of life

## Abstract

**Objectives:** Inflammatory bowel diseases (IBDs) are chronic inflammatory conditions of the gastrointestinal tract, comprising ulcerative colitis (UC) and Crohn’s disease (CD). Earlier onset of IBD symptoms has been linked to a higher prevalence of depression and anxiety. Evidence supports that cortisol abnormalities correlate with the development and severity of autoimmune diseases. The primary aim of this study was to investigate the correlation of morning salivary cortisol levels with self-reported mood (depression and anxiety) and quality of life in patients with IBD. **Methods:** This was a prospective, single-center study including outpatients with IBD. Enrolled patients provided a one-time morning salivary cortisol sample and electronically completed a one-time survey encompassing self-reported quality of life (Short Inflammatory Bowel Disease Questionnaire (SIBDQ)) and mood (Patient Health Questionnaire 8 (PHQ-8), General Anxiety Disorder-7 (GAD-7)). **Results:** A total of 36 patients (UC, n = 21) were included in the study. There was no correlation between morning salivary cortisol and depressive symptoms (PHQ-8: r = 0.007, *p* = 0.968) or quality of life (SIBDQ: r = −0.095, *p* = 0.606). However, there was a trend towards a positive correlation between self-reported anxiety symptoms by GAD-7 and salivary cortisol (r = 0.347, *p* = 0.052). A subgroup analysis showed a positive correlation between morning salivary cortisol and GAD-7 scores in patients with UC (r = 0.535, *p* = 0.015), but not in patients with CD (r = 0.064, *p* = 0.843). **Conclusions:** This pilot study is the first to associate cortisol with anxiety symptom severity in UC. Further research is needed to investigate the link between salivary cortisol, neuropsychiatric disease, and IBD outcomes.

## 1. Introduction

Inflammatory bowel diseases (IBDs) are chronic inflammatory conditions of the gastrointestinal tract, comprising ulcerative colitis (UC) and Crohn’s disease (CD). Patients with IBD may have intestinal and extra-intestinal complications including but not limited to diarrhea, rectal bleeding, fistulas, fatigue, arthralgias, and tenesmus [1]. IBD is often treated with immunosuppressive therapies, such as biologics, and surgery. The clinical manifestations and treatment modalities of IBD can have a lasting impact on patients’ mental health and quality of life, with some studies reporting a 15% prevalence of depressive symptoms [2,3,4,5].

There are high rates of comorbidity between gastrointestinal and neuropsychiatric diseases, whose primary physiologic link is likely the brain–gut axis [6,7,8]. Studies have shown that both organic and functional gastrointestinal disorders can be improved with psychiatric care [9,10]. Comorbid mental health disorders are one of the leading causes for excess costs related to patient care in IBD [11]. Active disease, changes in body function or appearance, and repeated episodes of perceived loss of control with each flare are thought to be major drivers of psychiatric comorbidity in the IBD patient population [12], and the subsequent emotional distress directly impacts quality of life [13]. Moreover, an earlier onset of IBD symptoms has been linked to a higher prevalence of neuropsychiatric disorders, specifically depression and anxiety [14]. However, there is no validated way to track the influence of the brain–gut axis in IBD outcomes. The melanocortin system is a complex set of molecular mediators and receptors that are involved in many physiological and homeostatic processes including the modulation of inflammatory processes such as IBD [15]. A recent study showed that the melanocortin 3 and 5 receptors were significantly more expressed in inflamed mucosa in the colon of both patients with CD and UC compared to normal mucosa, suggesting a potential use of these receptors in IBD pharmacology [16]. 

Evidence supports that early life stress and subsequent associated cortisol abnormalities correlate with the development and severity of autoimmune diseases including, for example, systemic lupus erythematosus, Sjogren’s syndrome, and systemic sclerosis [17]. This is thought to be mediated by the effects of both inflammation and stress on the hypothalamic–pituitary–adrenal (HPA) axis, making cortisol a potential biomarker in immune-mediated inflammatory disorders, like IBD. Moreover, cortisol has been shown to also be useful in predicting the likelihood of mental illness and impending onset and severity of symptoms [18]. In cardiovascular research, chronic stress measured from hair cortisol (levels over prior weeks) also correlates with established cardiometabolic risk factors for cardiovascular disease including high blood pressure, diabetes, and adiposity [19]. Salivary and hair cortisol levels of women with systemic lupus erythematosus, Sjogren’s syndrome, and systemic sclerosis were markedly higher and correlated with perceived stress and psychiatric testing when compared to healthy controls [17]. Similar data have been reported in multiple sclerosis [20]. However, little has been studied about cortisol’s significance in IBD.

There has been a surge in interest in finding effective biomarkers that inform providers on both physiologic stress and IBD outcomes [21,22,23]. In UC patients, perceived stress (from behavioral assessments, not surveys) significantly correlated with clinical flares defined as a simple clinical colitis activity index of 5 or higher, but only weakly correlated with biochemical markers including fecal calprotectin and morning salivary cortisol [21]. In patients with CD, a prospective study showed cognitive impairment and interleukin-6 abnormalities associated with significantly blunted cortisol awakening response (average of early morning cortisol samples after awakening), despite clinical remission [22].

This study aimed to investigate the correlation of morning salivary cortisol levels with self-reported mood (depression and anxiety) and quality of life in patients with IBD. A secondary objective was to investigate the association of salivary cortisol levels with biomarker or endoscopic remission.

## 2. Methods

### 2.1. Study Design and Population

In this prospective, single-center study, outpatients with IBD seen for routine care in the IBD clinic or infusion center between January 2023 and January 2024 were eligible. Patients were included in the study if they had a diagnosis of IBD, were at least 18 years old, and able to provide written consent and complete sample collection and questionnaire. Exclusion criteria included pregnancy, exercise within one hour of sample collection, shift work (i.e., alternating shift hour schedule, overnight shifts), comorbid hypertension, liver disease, heart disease, obesity (body mass index > 30), IBD-related surgery in the last 6 months, corticosteroid use within 6 months, active substance use, active malignancy, HPA axis-related issues (i.e., pituitary disease), oral mucosal bleeding at time of saliva collection, or smoking prior to saliva collection.

### 2.2. Salivary Sample Collection, Processing, and Cortisol Measurement

Enrolled patients provided a one-time morning salivary cortisol sample prior to 10:00 a.m. on the day of enrollment using Sarstedt’s SalivetteR Cortisol collection tubes. Awakening time was documented for each sample and salivary fluctuations were normalized to time since awakening (in hours). Samples were spun to isolate stimulated saliva and frozen in order to process in bulk at a later time. Once thawed, total protein in samples was determined by ultraviolet absorbance at 280 nm for normalization across samples. Saliva processing and cortisol level measurements were performed using Human Cortisol (Saliva) Human ELISA Kit (ab285353) using Mass Spec at 450 nm. The cross-reactivity of this ELISA assay is 6.8% for prednisolone, 4.22% for cortisone, and <0.1% for other hormones, including 11-deoxycortisol, estradiol, testosterone, and progesterone. A ratio of salivary cortisol levels to total protein was determined in order to standardize salivary cortisol quantities and is referred to as salivary cortisol levels.

### 2.3. Study Outcomes

Biomarker remission was defined as FC < 250 μg/g or CRP < 5 mg/L (for patients with both evaluations FC < 250 μg/g and CRP < 5 mg/dL) [24]. Endoscopic remission was defined as Simple Endoscopic Score for Crohn’s Disease (SES-CD) ≤2 for patients with CD and endoscopic Mayo score ≤1 for patients with UC [25]. Objective measures were assessed within 6 months from the salivary cortisol sample.

### 2.4. Survey Collection

Eligible patients electronically completed a one-time survey encompassing self-reported quality of life (Short Inflammatory Bowel Disease Questionnaire (SIBDQ)) and mood (Patient Health Questionnaire 8 (PHQ-8), General Anxiety Disorder-7 (GAD-7)) [26,27,28,29]. 

The SIBDQ is a quality of life instrument for community physicians managing IBD [26]. The patients are asked to choose and answer what best describes how they have felt over the past 2 weeks. Each item is scored by a 7-point graded scale, from 1 to 7, for an absolute score range from 10 (poor) to 70 (optimum) health-related quality of life. Each question can have the following answers (and respective scores): All of the time (1); Most of the time (2); A good bit of the time (3); Some of the time (4); A little of the time (5); Hardly any of the time (6); and None of the time (7). The questions of the SIBDQ are as follows: How often has the feeling of fatigue or of being tired and worn out been a problem for you during the last 2 weeks?; How often during the last 2 weeks have you had to delay or cancel a social engagement because of your bowel problem? How much difficulty have you had, as a result of your bowel problems, doing leisure or sports activities you would have liked to have done during the last 2 weeks? How often during the last 2 weeks have you been troubled by pain in the abdomen? How often during the last 2 weeks have you felt depressed or discouraged? Overall, in the last 2 weeks how much of a problem have you had with passing large amounts of gas? Overall, in the last 2 weeks how much of a problem have you had maintaining or getting to the weight you would like to be? How often during the last 2 weeks have you felt relaxed and free of tension? How much of the time during the last 2 weeks have you been troubled by a feeling of having to go to the bathroom even though your bowels were empty? How much of the time during the last 2 weeks have you felt angry as a result of your bowel problem? 

The PHQ8 quantifies the degree of depression severity [27]. The patients are asked to choose and answer that best describes how they have felt over the past 2 weeks. Patients with scores of 10 and over were found to be 91% sensitive and 74% specific for detecting major depressive disorder. Each question can have the following answers (and respective scores): Not at all (0); Several days (1); More than half the days (2); Nearly every day (3). The questions of the PHQ8 are as follows: Little interest or pleasure in doing things? Feeling down, depressed or hopeless? Trouble falling or staying asleep or sleeping too much? Feeling tired or having little energy? Poor appetite or overeating? Feeling bad about yourself—or that you are a failure or have let yourself or your family down? Trouble concentrating on things, such as reading the newspaper or watching TV? Moving or speaking so slowly that other people could have noticed? Or so fidgety or restless that you have been moving a lot more than usual? 

The GAD-7 is a screening tool and severity measure for general anxiety disorder [28]. The patients are asked to choose and answer what best describes how they have felt over the past 2 weeks. Patients with scores of 5, 10, and 15 are taken as cut-ff points for mild, moderate, and severe anxiety, respectively. Using the threshold score of 10, the GAD7 sensitivity is 89% and specificity is 82%. Each question can have the following answers (and respective scores): Not at all (0); Several days (1); More than half the days (2); Nearly every day (3). The questions of the PHQ8 are as follows: Feeling nervous, anxious or on edge? Not being able to stop or control worrying? Worrying too much about different things? Trouble relaxing; Being so restless that it is hard to sit still? Becoming easily annoyed or irritable? Feeling afraid as if something awful might happen? 

### 2.5. Statistical Analysis

Categorical variables were described as percentages and continuous variables were described as medians with interquartile range (IQR). Continuous variables were compared between groups using the Mann–Whitney U test. Categorical variables were compared between groups using the chi-square or the Fisher’s exact test, as appropriate. Correlation between salivary cortisol levels and SIBDQ, GAD-7. and PHQ-8 was evaluated using the Spearman’s rho test. A subgroup analysis was performed for patients with CD versus UC and for males versus females. A multivariate regression analysis to identified variables associated with the investigated outcomes was not conducted due to the small sample size. All analyses were performed using SPSS version 25.0 (SPSS, Chicago, IL, USA) and GraphPad Prism version 5.03 for Windows (GraphPad Software, San Diego, CA, USA). Statistical significance was set to a *p* value <0.05.

## 3. Results

### 3.1. Study Population

A total of 36 patients (UC, n = 21) were included in the study. The median age of the patients was 42 years, 56% were male, and 58% had a diagnosis of UC. The great majority of the patients (84%) received biological therapy. Patient demographic and clinical characteristics are shown in Table 1.

### 3.2. Study Outcomes

**Figure 1:**
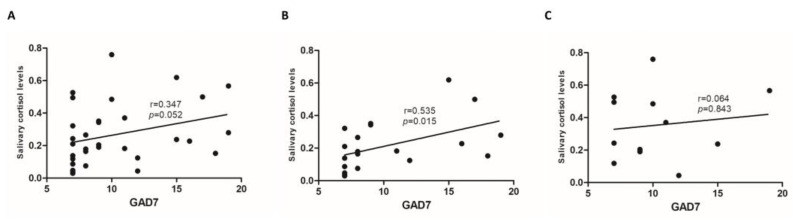
GAD-7 scores correlated to salivary cortisol (normalized to total salivary protein) in patients with inflammatory bowel disease (IBD) (**A**), ulcerative colitis (UC) (**B**), and Crohn’s disease (CD) (**C**). GAD: General anxiety disorder.

There was no correlation between morning salivary cortisol and depressive symptoms (PHQ-8: r = 0.007, *p* = 0.968) or quality of life (SIBDQ: r = −0.095, *p* = 0.606) in patients with IBD. However, there was a trend towards a positive correlation between self-reported anxiety symptoms by GAD-7 (r = 0.347, *p* = 0.052, Figure 1A) and salivary cortisol. A subgroup analysis based on the type of IBD, showed a positive correlation between morning salivary cortisol and GAD-7 scores in patients with UC (r = 0.535, *p* = 0.015, Figure 1B), but not in patients with CD (r = 0.064, *p* = 0.843, Figure 1C). 

There was no difference in salivary cortisol levels (median, (IQR)) between patients with CD and UC (0.243 (0.155–0.526) vs. 0.183 (0.106–0.333), respectively; *p* = 0.136) and there was no difference in GAD-7 (median, (IQR)) between patients with CD and UC (9.5 (7–11.8) vs. 8 (7–14.3), respectively; *p* = 0.780). Moreover, there was no difference in salivary cortisol levels (median, (IQR)) between male and female patients with IBD (0.240 (0.169–0.480) vs. 0.166 (0.087–0.414), respectively; *p* = 0.203). These results were consistent for both patients with CD and UC. 

Around two-thirds of the patients (65%) had biomarker remission, while around half of the patients (46%) had endoscopic remission. There was no statistical difference in cortisol levels (median, (IQR)) between patients with or without biomarker remission (0.282 (0.123–0.489) vs. 0.200 (0.142–0.259) respectively; *p* = 0.471) or endoscopic remission (0.273 (0.159–0.364) vs. 0.196 (0.084–0.517), respectively; *p* = 0.877). These results were consistent for both patients with CD and UC.

## 4. Discussion

Cumulative data suggest that anxiety and depression are common comorbidities in patients with IBD [30]. A retrospective analysis using an IBD natural history registry from a single tertiary care referral center including 432 patients with IBD showed that anxiety and depression are common in the setting of IBD (44.4%) and are strongly associated with higher prescription of corticosteroids and biological therapy as well as utilization of healthcare resources (emergency department visits and hospitalization) [30]. Holistic and multidisciplinary care improves outcomes, but we lack validated, objective measurements to track how perceived stress, depression, anxiety, and low quality of life impact IBD flares. Patients with IBD may also have reduced physical activity, partly due to fears that exercise might negatively impact their disease, as shown by the BE-FIT-IBD studies [31,32]. Our study found no statistically significant correlations between objective outcomes (serum CRP, FC, endoscopic scores) and quality of life, mood, or anxiety survey data (PHQ-8, GAD-7, and SIBDQ) with morning salivary cortisol in patients with IBD.

Interestingly, a subgroup analysis did show a positive correlation between morning salivary cortisol and GAD-7 scores in patients with UC. However, our data did not clarify if higher cortisol levels in the UC cohort influenced therapeutic outcomes, as these levels did not correlate with higher CRP, FC, or endoscopic activity. The same trend was not seen in CD altogether, possibly due to lower representation in our cohort. Alternatively, unique microbiome and metabolomic signatures in UC patients could have played a role in this trend but these variables were not measured. Yuan and colleagues have identified unique microbiome and metabolomic signatures in UC patients with depression and anxiety, which differ from UC patients without psychiatric comorbidity [27]. Patients with UC and depression/anxiety compared to those without had lower fecal microbial community richness and diversity, with more *Lactobacillales*, *Sellimonas*, *Streptococcus*, and *Enterococcus* but less *Prevotella_9* and *Lachnospira* [33]. 

Our study was limited by the rather small sample size, especially for CD. Moreover, the timing of cortisol sample collection, particularly in relation to steroid use (excluded from this study) which requires further investigation. Future studies should consider home salivary kits for more frequent data collection, following the same patient through active disease and remission, all of which would potentially yield more definitive findings on the potential clinical use for monitoring salivary cortisol. Finally, the great majority (84%) of the patients received biologic therapy which could potentially impact the results. 

Ultimately, our data serve as a proof of concept, indicating that larger studies may find significant links between salivary cortisol, neuropsychiatric disease (i.e., GAD-7), and IBD outcomes. This pilot study is the first to associate cortisol with anxiety symptom severity in UC. Further research into the role of salivary cortisol and other HPA axis markers as predictors of IBD activity and mental health stress has the potential for enabling healthcare providers to intervene early and potentially improve overall outcomes in IBD.

## Figures and Tables

**Table 1:** Patient demographic and clinical characteristics.

Patient Characteristics	N = 36
Male, (%)	20 (56)
Age, median (IQR), years	42 (28–50)
IBD type, (%)	
CD	15 (42)
UC	21 (58)
Biological therapy, (%)	27/32 (84)
GAD-7, median (IQR)	9 (7–12)
PHQ8, median (IQR)	12 (11–15)
SIBDQ, median (IQR)	52 (57–61)
Biomarker remission, (%)	22/34 (65)
Endoscopic remission, (%)	12/26 (46)

IBD: inflammatory bowel disease; CD: Crohn’s disease; UC: ulcerative colitis; IQR: interquartile range; GAD: general anxiety disorder; SIBDQ: Short Inflammatory Bowel Disease Questionnaire; PHQ: Patient Health Questionnaire.

## Data Availability

The data that support the findings of this study can be made available upon reasonable request.
